# Association of the Transmembrane Protease Serine 6 rs855791 Variant and Nongenetic Factors With Iron Deficiency Among Female Medical Students in Yogyakarta: Protocol for a Case-Control Study

**DOI:** 10.2196/79555

**Published:** 2025-10-29

**Authors:** Fenty Fenty, Erna Kristin, Tri Ratnaningsih, Supanji Supanji, Jajah Fachiroh, Dwi Aris Agung Nugrahaningsih

**Affiliations:** 1 Faculty of Medicine, Public Health and Nursing Universitas Gadjah Mada Yogyakarta Indonesia; 2 Faculty of Pharmacy Sanata Dharma University Yogyakarta Indonesia; 3 Department of Pharmacology and Therapy Faculty of Medicine, Public Health and Nursing Universitas Gadjah Mada Yogyakarta Indonesia; 4 Department of Clinical Pathology and Medical Laboratory Faculty of Medicine, Public Health and Nursing Universitas Gadjah Mada Yogyakarta Indonesia; 5 Department of Opthalmology Faculty of Medicine, Public Health and Nursing Universitas Gadjah Mada Yogyakarta Indonesia; 6 Department of Histology & Cell Biology Faculty of Medicine, Public Health and Nursing Universitas Gadjah Mada Yogyakarta Indonesia

**Keywords:** transmembrane protease serine 6, rs855791, TMPRSS6, rs855791, nongenetic, iron deficiency, female, women, medical students

## Abstract

**Background:**

Iron deficiency is the most widespread nutritional deficiency worldwide, and it is the primary cause of anemia, particularly in low- and middle-income countries such as Indonesia. Iron deficiency has a multifactorial etiology involving complex interactions between genetic factors, especially the transmembrane protease serine 6 (*TMPRSS6)* rs855791 variant, which encodes matriptase-2, a protein involved in regulating hepcidin expression, and nongenetic factors, including sociodemographics, nutritional status, iron intake, and menstrual patterns. Women of reproductive age, including medical students, are susceptible to iron deficiency owing to unhealthy dietary habits, growth requirements, and menstruation. Iron deficiency among medical students may lead to decreased academic performance and productivity. Additionally, as future mothers, women may experience a heightened risk of delivering children with intellectual disabilities and various psychosocial impairments. Owing to the significant consequences of this condition, identifying the underlying causes of iron deficiency is crucial. The high prevalence of iron deficiency in Indonesia poses a challenge in addressing these contributing factors to effectively reduce its occurrence.

**Objective:**

This study aims to investigate the association of the *TMPRSS6* rs855791 variant and nongenetic factors with iron deficiency among female medical students in Yogyakarta, Indonesia.

**Methods:**

This is a case-control study. We will recruit female medical students from the Faculty of Medicine, Public Health, and Nursing of Universitas Gadjah Mada in Yogyakarta, Indonesia. The inclusion criteria are being a final-year female undergraduate medical student who has not entered the clinical clerkship phase, aged 18 to 24 years, not pregnant, providing written consent, and having no history of chronic and inflammatory diseases, congenital diseases, hematological disorders, or blood transfusions during the last 3 months. Participants will be excluded if the C-reactive protein level is higher than 5 mg/L. Participants will be further grouped according to iron status criteria. Profiles of hemogram and iron markers will be compared between the case and control groups using the independent samples 2-tailed *t* test or the Mann-Whitney *U* test, while genotype and allele frequencies will be analyzed using the chi-square test. One-way ANOVA or the Kruskal-Wallis test will be used to assess the impact of different genotypes on iron marker levels. Multivariate analysis will be performed with logistic regression to determine factors independently associated with iron deficiency risk. *P*≤.05 will be considered statistically significant.

**Results:**

The study received funding in January 2025. Data collection began in February 2025 and is anticipated to conclude by October 2025. At the time of manuscript submission, 115 participants had been enrolled. The study findings are expected to be published in 2026.

**Conclusions:**

This study will determine the interaction between the *TMPRSS6* rs855791 variant and nongenetic factors that contribute to the risk of iron deficiency among female medical students in Yogyakarta, Indonesia.

**International Registered Report Identifier (IRRID):**

DERR1-10.2196/79555

## Introduction

### Background

Iron deficiency is the most prevalent nutritional deficiency globally. It accounts for more than 60% of anemia cases [1,2]. This condition generally affects children younger than 5 years, pregnant women, and women of reproductive age, especially those in low- and middle-income countries (LMICs) [3,4]. The World Health Organization reports that anemia is most widespread in Africa and Southeast Asia [4]. A study conducted across 7 South and Southeast Asian countries found that more than half of women of reproductive age were affected by anemia [5]. Another study, from the Demographic and Health Survey of 46 LMICs, showed that the prevalence of anemia in pregnant (45.2%) and nonpregnant women (39.5%) was higher than the global prevalence [6]. According to the 2018 Indonesian Basic Health Research survey, the overall prevalence of anemia in Indonesia was 23.7% (women: 27.2%; men: 20.3%) [7].

Iron plays a crucial role in several essential biological processes in the body, including electron transfer, oxygen transport, cell growth, aerobic metabolism, DNA synthesis, and enzymatic processes, and functions in muscle and neurological development, nerve fiber myelination, and neurotransmitter production [8-10]. Iron deficiency can affect cognitive development, school achievements, work performance, and productivity [11-13]. Iron deficiency that occurs during essential phases of brain development can cause permanent structural damage [9]. Iron deficiency during pregnancy can result in detrimental effects on infants and children. Many women of reproductive age enter pregnancy with low iron levels and may have no iron reserves whatsoever [2,14].

Several factors contribute to iron deficiency, including environmental factors, nutritional deficiencies, iron intake, sociodemographics, social habits, diet, lifestyle, infections, and blood loss (eg, menstrual bleeding or childbirth among women) [6,15,16]. Studies have also reported the role of genetic variability in an individual’s iron status, accounting for approximately 20% to 30% of the variation [17-21]. Genome-wide association studies (GWASs) have demonstrated that single-nucleotide polymorphisms (SNPs) in certain genes are involved in hepcidin hormone regulation pathways that control iron homeostasis. Among these genes, loci on the transmembrane protease serine 6 (*TMPRSS6*) gene are frequently linked to low levels of iron and hematological indices. The most common SNP in the *TMPRSS6* gene that is strongly associated with iron deficiency anemia is rs855791. This gene encodes matriptase-2, which interacts with and promotes the cleavage of hemojuvelin on the cell surface, leading to the release of soluble hemojuvelin and inhibiting hepcidin expression [17,18,21,22]. This gene polymorphism is linked to a reduced ability to decrease hepcidin production, thereby causing increased levels in the circulation despite iron deficiency. Furthermore, matriptase-2 dysfunction is associated with iron-refractory iron deficiency anemia [17,21,23].

Iron deficiency has a multifactorial etiology involving complex interactions between genetic and nongenetic factors. Although GWASs have identified risk alleles for complex genetic characteristics related to iron status, the detected risk alleles did not fully explain heritability. Moreover, the impact of allele frequency and biological adaptation varies across ethnicities owing to environmental and physiological factors. This complexity presents a challenge in effectively addressing the contributing factors of iron deficiency in the population [17,21].

In Indonesia, research on the association of genetic variations and nongenetic factors with iron deficiency remains limited, particularly in vulnerable populations, including female medical students, who are part of the representative population of women of reproductive age. They are susceptible to the risk of developing iron deficiency. Moreover, this group is particularly relevant to study owing to its more homogeneous characteristics. Despite having good knowledge of preventing iron deficiency anemia, they frequently practice unhealthy eating habits, including skipping breakfast, snacking, consuming fast food, and lacking fruit and vegetable intake, and unhealthy lifestyle habits, such as not engaging in physical activity due to heavy study loads and extracurricular activity schedules in college. Furthermore, they experience physiological menstrual blood loss and remain in a growth period that requires adequate nutritional intake [24,25].

### This Study

Several studies in India, Pakistan, Iran, and Saudi Arabia have reported a significant rate of iron deficiency and anemia among female medical students, ranging from 16% to 63%, especially among those who live in dormitories and have a low or high BMI [24-29]. However, in Indonesia, only 1 study on female medical students with a sufficient socioeconomic background revealed a relatively low anemia prevalence of 13.6% [30]. The approach to managing iron deficiency has yet to fully consider genetic and nongenetic factors. Consequently, despite efforts to promote the distribution of iron supplements, the prevalence of iron deficiency remains high. Identifying the primary determinants of iron deficiency is crucial for more efficient management strategies. By recognizing these determinants, efforts can be made to increase the body’s iron reserves and provide appropriate prevention and treatment. Identifying these factors is also valuable in preparing adolescent girls for pregnancy to prevent and reduce the risk of maternal mortality, preterm delivery, low birth weight, and stunting. Therefore, our study aims to analyze the association of the *TMPRSS6* rs855791 variant and nongenetic factors with iron deficiency in apparently healthy female medical students.

## Methods

### Study Design and Setting

This study will use a case-control design. Cases and controls will consist of medical students at the Faculty of Medicine, Public Health, and Nursing, Universitas Gadjah Mada in Yogyakarta, Indonesia. Participants will be selected using purposive sampling according to predefined inclusion and exclusion criteria. The study will specifically target final-year female undergraduate medical students (aged 18-24 years) before entering the clinical clerkship phase; who have provided written informed consent; are not pregnant; and have no history of chronic and inflammatory diseases, congenital diseases, hematological disorders, or blood transfusions within the previous 3 months. Participants with elevated levels of the inflammatory biomarker C-reactive protein (CRP; elevated level defined as >5 mg/L) will be excluded. Study participants will be classified into the control and case groups based on their serum ferritin (SF) and hemoglobin levels. A case-control ratio of 1:1 will be applied. The matching condition will be an age difference of less than 3 years. The control group will include participants with normal hemoglobin (≥12 g/dL) and SF (≥30 μg/L) levels. The case group will include participants with iron deficiency. Iron deficiency will be determined based on SF levels of less than 30 μg/L with or without anemia [10,31].

### Sample Size

This study will be conducted from February 2025 to January 2026. The sample size will be determined using the unpaired categorical analytical research sample size formula with a confidence level of 95%, α of 5%, and research power of 80% (with β of 20%). The sample size calculation will use the proportion of exposed controls and the odds ratio of each independent variable for the risk of iron deficiency, yielding a minimum required sample size of 116 participants (58 cases and 58 controls). This sample size will ensure adequate statistical power to examine the influence of sociodemographic characteristics (type of residence and parental income), BMI, menstrual pattern, iron intake, and the *TMPRSS6* rs855791 genetic variant on the risk of iron deficiency.

### Data Collection and Analysis

We will use questionnaires for collecting data on the demographics, medical history, and socioeconomic information of the participants. The dietary iron intake of the participants will be assessed using the IRON Intake Calculation-Food Frequency Questionnaire, which has been modified and evaluated for validity and reliability [32]. This questionnaire contains information about the primary sources of iron, which are divided into 12 food groups, including meat, processed meat, eggs, fish, dairy products, cereal products, fruits, vegetables, tubers, oils, nuts, and chocolate products. For each food group included in the questionnaire, participants will be asked to report portion sizes and consumption frequency over the preceding week. The total weekly iron intake calculated from these responses will then be divided by 7 to estimate the mean daily iron intake. The NutriSurvey software will be used for analyzing iron intake [32,33]. Participants’ nutritional status will be measured using BMI. Menstrual patterns will be evaluated using a menstrual pictogram for 2 menstrual cycles. Menorrhagia is defined as menstrual bleeding exceeding 80 mL for each cycle [34,35].

Laboratory investigations will be performed as mentioned subsequently. In total, 7 mL of venous blood will be collected under aseptic conditions between 8 AM and 11 AM using 2 different tubes. A total of 3 mL of blood will be collected from each participant using an ethylenediaminetetraacetic acid tripotassium salt tube, with 2 mL of blood allocated for hemogram and reticulocyte profile analysis and 1 mL for genotyping. All laboratory analyses will be performed in accredited laboratories, strictly following the manufacturer’s instructions and under Good Clinical and Laboratory Practice conditions with internal quality controls to ensure accuracy and reliability. The hemogram and reticulocyte profile will be analyzed within 1 hour following sample collection using an automated hematology analyzer, Mindray BC-760 (Mindray Corporation). The remaining 4 mL will be collected into plain gel separator tubes and subsequently centrifuged. Serum samples will be stored at –20 °C for CRP and SF examinations and at –80 °C for serum hepcidin examinations. A cobas c 111 analyzer (particle-enhanced immunoturbidimetric assay; Roche Diagnostics), a VIDAS analyzer (enzyme-linked fluorescent assay; bioMérieux), and a sandwich human enzyme-linked immunosorbent assay kit (catalog number EH3221; Wuhan Fine Biotech Co, Ltd) will be used for CRP, SF, and serum hepcidin measurement.

### Genotyping Procedure

Genomic DNA will be isolated from 1 mL of ethylenediaminetetraacetic acid–anticoagulated whole blood using a commercially available DNA extraction kit (gSYNC DNA Blood Mini kit; catalog number 51104; Geneaid Biotech Ltd), following the manufacturer’s protocol. The concentration of the extracted DNA will be assessed using a NanoDrop spectrophotometer (MaestroGen) to assess the A260/A280 and A260/230 absorbance ratios. All extracted DNA samples will be preserved at –80 °C. *TMPRSS6* genotyping for the rs855791 polymorphism will be conducted using the TaqMan genotyping assay protocol (Applied Biosystems). PCR will be performed using a Taq DNA polymerase kit following the manufacturer’s instructions.

### Data Analysis Plan

The collected data will be categorized into quantitative and qualitative data. SPSS Statistics (version 22; IBM Corp) will be used for the analysis. The Kolmogorov-Smirnov test will be used to assess whether the data follow a normal distribution. If the data are normally distributed, quantitative data will be presented as means and SDs; if not, the data will be presented as medians (minimum and maximum). Qualitative data will be presented as frequencies and percentages.

Parameters measuring hemogram profiles and iron status will be compared between the case and control groups using the independent samples 2-tailed *t* test or Mann**-**Whitney *U* test. Differences in genotype and allele frequencies between the case and control groups will be evaluated using the chi-square test. To determine the effects of individual SNPs on iron markers, comparison of iron status across the genotyping classes (AA, AG, and GG) will be performed using 1-way ANOVA or the Kruskal-Wallis test. Assessment of bivariate associations between each independent variable and the outcome (dependent variable) will be conducted using the chi-square or Fisher test. Risk factors with a *P* value of <.25 in the bivariate analysis will be further analyzed through multivariate logistic regression analysis.

All risk factors meeting the aforementioned selection criteria will be entered one by one in the multivariate analysis, starting with the most significant factors from the bivariate analysis. The selection of the final model equation will be based on parsimony, biological interpretability, and statistical significance (*P*≤.05). This final model equation will be assessed using the Hosmer-Lemeshow test and the area under the curve value obtained through receiver operating characteristic analysis. Subsequently, the results of this final equation will be used for predicting the probability of iron deficiency.

### Ethical Considerations

The study protocol has been formally approved by the medical and health research ethics committee of the Faculty of Medicine, Public Health and Nursing, Universitas Gajah Mada in Yogyakarta, Indonesia (KE/FK/0114/EC/2025). Prior to enrollment, written informed consent will be obtained from all participants after they have been fully informed about the study objectives, procedures, potential risks and benefits, and the voluntary nature of their participation. To ensure confidentiality, all collected data will be securely stored with access restricted to authorized members of the research team. As a token of appreciation for their time, participants will receive a snack following the blood draw and reimbursement of Rp 50,000 (US $3.02) for transportation costs.

## Results

Data collection commenced in February 2025 and is expected to conclude by October 2025. Data analysis is scheduled for completion by the end of 2025, with the publication of study findings anticipated in 2026.

## Discussion

### Anticipated Findings

Anemia remains a prevalent health issue in LMICs, with iron deficiency identified as its primary cause. Most anemia cases are preventable and manageable, provided that the underlying causes are identified. Consequently, recognizing the multifactorial etiology of anemia is crucial for developing effective and tailored therapeutic strategies [36]. We anticipate that this study will identify both genetic variations and nongenetic factors impacting the risk of iron deficiency.

Several GWASs have consistently demonstrated a strong correlation between iron status and variants in the *TMPRSS6* gene, particularly the SNP rs855791, across various populations, predominantly in Asia. Despite the significant burden of iron deficiency in Asia, particularly in Southeast Asian nations such as Indonesia, studies focusing on unique gene variants associated with iron homeostasis in humans remain limited [18,21].

Studies by Hamed et al [37] and Elmahdy et al [38] revealed that individuals with iron deficiency or iron deficiency anemia exhibited a significantly increased frequency of the rs855791 variant (both heterozygous and homozygous) compared with the control group. Conversely, Jallow et al [20] and Varikuti et al [39] did not observe any association between this variant and the iron status. Shinta et al [19] investigated children aged 12 to 17 months in the Sasak community of East Lombok regency, West Nusa Tenggara province, Indonesia, and reported that iron intake had a stronger influence on SF levels than the rs855791 polymorphism. Building upon these findings, our study is anticipated to provide further evidence regarding the prevalence and potential role of the rs855791 gene variant in the Indonesian population, particularly among female medical students.

Research results from LMICs have indicated that nongenetic factors, including poor socioeconomic status, illiteracy, lack of formal education, an interpregnancy interval of less than 2 years, excessive menstrual bleeding, living in rural areas, the lack of physical activity, poor nutritional intake, low BMI, obesity, and insufficient knowledge regarding anemia, can all contribute to anemia, particularly iron deficiency–induced anemia [16,25,27,28,36,37]. Most existing research has focused on isolated risk factors, either genetic or nongenetic, with limited investigation into their combined effects. Only a few studies have explored the interaction between genetic factors and a single nongenetic factor. Shinta et al [19] investigated the relationship between *TMPRSS6* gene polymorphism and iron intake on the risk of iron deficiency, whereas Pei et al [22] reported that certain genetic variations have a protective role against iron deficiency anemia among women with menorrhagia. The occurrence of iron deficiency involves a multifactorial interaction between genetic and nongenetic determinants. We aim to identify the interplay between these factors and susceptibility to iron deficiency in the specific context of female medical students. A comprehensive understanding of both genetic and nongenetic contributors will support the development of targeted and evidence-based therapeutic approaches informed by the identified causal factors.

### Strengths and Limitations

A notable strength of this study lies in its comprehensive methodology for investigating both genetic and nongenetic factors associated with iron deficiency among female medical students. Iron intake, nutritional status, menstrual patterns, and sociodemographic characteristics will be the nongenetic variables assessed, whereas the genetic aspect will focus on the rs855791 variant. In addition, serum hepcidin levels, a key regulator of iron homeostasis associated with the rs855791 variant and other iron-related biomarkers, will be measured. Despite these strengths, several limitations should be acknowledged. First, hemoglobin electrophoresis will not be performed in the case group, thereby limiting the ability to detect coexisting conditions, such as thalassemia trait, in participants with iron deficiency. Second, this study will not include measurements of vitamin D levels. Given vitamin D’s role in regulating hepcidin expression, its deficiency may lead to elevated hepcidin levels, thereby reducing iron bioavailability and contributing to iron deficiency [40,41]. Third, the recruitment of participants from a single academic institution may limit the external validity of the findings, potentially constraining their generalizability to the broader population of women in Indonesia. Nevertheless, the conceptual framework and integrated methodological approach addressing both genetic and nongenetic factors may meaningfully contribute to community-based iron deficiency screening strategies, particularly for young, educated women in urban areas.

Further research is recommended to address this study’s limitations, including the assessment of hemoglobin electrophoresis and vitamin D levels, which may affect the accuracy of iron deficiency evaluation, by expanding recruitment to a larger, multicenter scale. Moreover, subsequent studies should investigate the efficacy of treatment approaches for iron deficiency based on the causal determinants identified in this study.

### Conclusions

Our study aims to elucidate how genetic and nongenetic factors contribute to the risk of iron deficiency in women of reproductive age, especially female medical students in Indonesia. The results are expected to provide valuable insights into the primary determinants contributing to iron deficiency in this population.

## Abbreviations

CRP: C-reactive protein
GWAS: genome-wide association study
LMIC: low- and middle-income country
SF: serum ferritin
SNP: single-nucleotide polymorphism
TMPRSS6: transmembrane protease serine 6

## References

[1] Anaemia in women and children. World Health Organization.

[2] Derman RJ, Patted A (2023). Overview of iron deficiency and iron deficiency anemia in women and girls of reproductive age. Int J Gynaecol Obstet.

[3] Kassebaum NJ, Jasrasaria R, Naghavi M, Wulf SK, Johns N, Lozano R, Regan M, Weatherall D, Chou DP, Eisele TP, Flaxman SR, Pullan RL, Brooker SJ, Murray CJ (2014). A systematic analysis of global anemia burden from 1990 to 2010. Blood.

[4] (2023). Accelerating anaemia reduction: a comprehensive framework for action. World Health Organization.

[5] Sunuwar DR, Singh DR, Chaudhary NK, Pradhan PM, Rai P, Tiwari K (2020). Prevalence and factors associated with anemia among women of reproductive age in seven South and Southeast Asian countries: evidence from nationally representative surveys. PLoS One.

[6] Alem AZ, Efendi F, McKenna L, Felipe-Dimog EB, Chilot D, Tonapa SI, Susanti IA, Zainuri A (2023). Prevalence and factors associated with anemia in women of reproductive age across low- and middle-income countries based on national data. Sci Rep.

[7] Kemenkes RI (2019). Laporan Nasional Riskesdas 2018. Health Research and Development Agency, Government of Indonesia.

[8] Yehuda S, Mostofsky DI (2010). Iron Deficiency and Overload: From Basic Biology to Clinical Medicine.

[9] Georgieff MK (2023). The importance of iron deficiency in pregnancy on fetal, neonatal, and infant neurodevelopmental outcomes. Int J Gynaecol Obstet.

[10] MacLean B, Sholzberg M, Weyand AC, Lim J, Tang G, Richards T (2023). Identification of women and girls with iron deficiency in the reproductive years. Int J Gynaecol Obstet.

[11] Jung J, Rahman MM, Rahman MS, Swe KT, Islam MR, Rahman MO, Akter S (2019). Effects of hemoglobin levels during pregnancy on adverse maternal and infant outcomes: a systematic review and meta-analysis. Ann N Y Acad Sci.

[12] Teressa H (2019). A review on major causes of anemia and its prevention mechanism. Int J Cell Sci Mol Biol.

[13] Marcus H, Schauer C, Zlotkin S (2021). Effect of anemia on work productivity in both labor- and nonlabor-intensive occupations: a systematic narrative synthesis. Food Nutr Bull.

[14] (2001). Iron deficiency anaemia: assessment, prevention, and control. A guide for programme managers. World Health Organization.

[15] Nainggolan O, Hapsari D, Titaley CR, Indrawati L, Dharmayanti I, Kristanto AY (2022). The relationship of body mass index and mid-upper arm circumference with anemia in non-pregnant women aged 19-49 years in Indonesia: analysis of 2018 Basic Health Research data. PLoS One.

[16] Sappani M, Mani T, Asirvatham ES, Joy M, Babu M, Jeyaseelan L (2023). Trends in prevalence and determinants of severe and moderate anaemia among women of reproductive age during the last 15 years in India. PLoS One.

[17] Gichohi-Wainaina WN, Melse-Boonstra A, Swinkels DW, Zimmermann MB, Feskens EJ, Towers GW (2015). Common variants and haplotypes in the TF, TNF-α, and TMPRSS6 genes are associated with iron status in a female Black South African population. J Nutr.

[18] Jallow MW, Cerami C, Clark TG, Prentice AM, Campino S (2020). Differences in the frequency of genetic variants associated with iron imbalance among global populations. PLoS One.

[19] Shinta D, Adhiyanto C, Htet MK, Fahmida U, Asmarinah (2019). The association of TMPRSS6 gene polymorphism and iron intake with iron status among under-two-year-old children in Lombok, Indonesia. Nutrients.

[20] Jallow MW, Campino S, Prentice AM, Cerami C (2021). Association of common TMPRSS6 and TF gene variants with hepcidin and iron status in healthy rural Gambians. Sci Rep.

[21] Mohd Atan FN, Wan Mohd Saman WA, Kamsani YS, Khalid Z, Abdul Rahman A (2022). TMPRSS6 gene polymorphisms associated with iron deficiency anaemia among global population. Egypt J Med Hum Genet.

[22] Pei SN, Ma MC, You HL, Fu HC, Kuo CY, Rau KM, Wang MC, Lee CT (2014). TMPRSS6 rs855791 polymorphism influences the susceptibility to iron deficiency anemia in women at reproductive age. Int J Med Sci.

[23] Yılmaz Keskin E, Yenicesu (2015). Iron-refractory iron deficiency anemia. Turk J Haematol.

[24] Pandey S, Singh A (2022). A cross sectional study of nutritional anemia among medical students in a medical college, at Bilaspur, Chhattisgarh. Natl J Med Res.

[25] Jawed S, Tariq S, Tariq S, Kamal A (2017). Frequency of nutritional anemia among female medical students of Faisalabad. Pak J Med Sci.

[26] Shams S, Asheri H, Kianmehr A, Ziaee V, Koochakzadeh L, Monajemzadeh M, Nouri M, Irani H, Gholami N (2010). The prevalence of iron deficiency anaemia in female medical students in Tehran. Singapore Med J.

[27] Vibhute NA, Shah U, Belgaumi U, Kadashetti V, Bommanavar S, Kamate W (2019). Prevalence and awareness of nutritional anemia among female medical students in Karad, Maharashtra, India: a cross-sectional study. J Family Med Prim Care.

[28] Alkhaldy HY, Hadi RA, Alghamdi KA, Alqahtani SM, Al Jabbar IS, Al Ghamdi IS, Bakheet OS, Saleh RA, Shehata SF, Aziz S (2020). The pattern of iron deficiency with and without anemia among medical college girl students in high altitude southern Saudi Arabia. J Family Med Prim Care.

[29] Hamed M, Zaghloul A, Halawani SH, Fatani BA, Alshareef B, Almalki A, Alsharif E, ALhothaly QA, Alhadhrami S, Abd Elmoneim HM (2024). Prevalence of overweight/obesity associated with anemia among female medical students at Umm Al-Qura University in Makkah, Saudi Arabia: a cross-sectional study. Cureus.

[30] Wratsangka R, Tungka EX, Murthi AK, Ali S, Nainggolan M, Sahiratmadja E (2024). Anemia among medical students from Jakarta: Indonesia-iron deficiency or carrier thalassemia?. Anemia.

[31] Kumar A, Sharma E, Marley A, Samaan MA, Brookes MJ (2022). Iron deficiency anaemia: pathophysiology, assessment, practical management. BMJ Open Gastroenterol.

[32] Ayuningtyas IN, Tsani AF, Candra A, Dieny FF (2022). Analisis asupan zat besi heme dan non heme, vitamin B12 dan folat serta asupan enhancer dan inhibitor zat besi berdasarkan status anemia pada santriwati. J Nutr Coll.

[33] Głąbska D, Guzek D, Ślązak J, Włodarek D (2017). Assessing the validity and reproducibility of an iron dietary intake questionnaire conducted in a group of young Polish women. Nutrients.

[34] Magnay JL, O'Brien S, Gerlinger C, Seitz C (2020). Pictorial methods to assess heavy menstrual bleeding in research and clinical practice: a systematic literature review. BMC Womens Health.

[35] Magnay JL, O'Brien S, Gerlinger C, Seitz C (2018). A systematic review of methods to measure menstrual blood loss. BMC Womens Health.

[36] Osborn AJ, Muhammad GM, Ravishankar SL, Mathew AC (2021). Prevalence and correlates of anemia among women in the reproductive age (15-49 years) in a rural area of Tamil Nadu: an exploratory study. J Educ Health Promot.

[37] Hamed HM, Bostany EE, Motawie AA, Abd Al-Aziz AM, Mourad AA, Salama HM, Kamel S, Hassan EM, Helmy NA, El-Saeed GS, Elghoroury EA (2024). The association of TMPRSS6 gene polymorphism with iron status in Egyptian children (a pilot study). BMC Pediatr.

[38] Elmahdy M, Elhakeem H, Gaber F, Mourad F (2018). TMPRSS6 gene polymorphism and serum hepcidin in iron deficiency anemia. Egypt J Hosp Med.

[39] Varikuti SR, Parasannavar DJ, Rajkumar H, Bhukya T, Satyanarayana U, Kumar M (2021). The role of gene variants in the iron metabolism of anemic adolescent girls. Cureus.

[40] Mogire RM, Muriuki JM, Morovat A, Mentzer AJ, Webb EL, Kimita W, Ndungu FM, Macharia AW, Cutland CL, Sirima SB, Diarra A, Tiono AB, Lule SA, Madhi SA, Prentice AM, Bejon P, Pettifor JM, Elliott AM, Adeyemo A, Williams TN, Atkinson SH (2022). Vitamin D deficiency and its association with iron deficiency in African children. Nutrients.

[41] Seong JM, Park CE, Gi MY, Cha JA, Moon AE, Lee JH, Sung HH, Lim JH, Oh SH, Chung CH, Seo EK, Yoon H (2021). Gender difference in the relationship between anemia and vitamin D in Korean adults: the fifth Korea National Health and Nutrition Examination Survey. J Clin Biochem Nutr.

